# Assessment of Screening for Nasal Obstruction among Sleep Dentistry Outpatients with Obstructive Sleep Apnea

**DOI:** 10.3390/dj8040119

**Published:** 2020-10-14

**Authors:** Arisa Sawa, Hiroshi Suzuki, Hideo Niwa, Sumito Oguchi, Tatsuo Yagi, Yoshihiro Iwata, Yasuhide Makiyama, Chin Moi Chow, Osamu Komiyama

**Affiliations:** 1 Division of Oral Health Science, Department of Oral Function and Rehabilitation, Nihon University School of Dentistry at Matsudo, Matsudo, Chiba 271-8587, Japan; maar17004@g.nihon-u.ac.jp (A.S.); iwata.yoshihiro@nihon-u.ac.jp (Y.I.); komiyama.osamu@nihon-u.ac.jp (O.K.); 2 Department of Head and Neck Surgery, Nihon University School of Dentistry at Matsudo, Matsudo, Chiba 271-8587, Japan; niwa.hideo@nihon-u.ac.jp (H.N.); makiyama.yasuhide@nihon-u.ac.jp (Y.M.); 3 Department of Internal Medicine, Nihon University School of Dentistry at Matsudo, Matsudo, Chiba 271-8587, Japan; oguchi.sumito@nihon-u.ac.jp; 4 Faculty of Law, Seiwa University, Kisarazu City, Chiba 292-8555, Japan; yagitatsuo@gmail.com; 5 Sleep Research Group, Charles Perkins Centre, University of Sydney, Sydney 2006, Australia; chin-moi.chow@sydney.edu.au; 6 Sydney School of Health Sciences, Faculty of Medicine and Health, University of Sydney, Sydney 2006, Australia

**Keywords:** apnea–hypopnea index, Mallampati classification, mandibular advancement devices, nasal obstruction, sleep apnea, obstructive

## Abstract

Oral appliances (OA), a common treatment modality for obstructive sleep apnea (OSA), are not suitable for patients with nasal obstruction. Rhinomanometry, the gold standard technique to assess nasal airway resistance, is not readily available in sleep dentistry clinics. We demonstrate the use of a portable lightweight peak nasal inspiratory flow (PNIF) rate meter to objectively assess nasal airflow and utilized the Nasal Obstruction Symptom Evaluation (NOSE) scale to subjectively assess nasal obstruction in 97 patients with OSA and 105 healthy controls. We examined the correlations between the following variables between the groups: demographics, body mass index, PNIF, NOSE scale scores, apnea–hypopnea index (AHI), minimum SpO_2_ (SpO_2_min), Mallampati classification, and Epworth Sleepiness Scale (ESS) scores. Patients with OSA had significantly lower PNIF values and higher NOSE scores than controls. In the patient group, PNIF was not significantly correlated with AHI, SpO_2_min, Mallampati classification, or NOSE or ESS scores. Lower PNIF values and higher NOSE scores suggested impaired nasal airflow in the OSA group. As daytime PNIF measurement bears no relationship to AHI, this cannot be used alone in predicting the suitability of treatment for OSA with OA but can be used as an adjunct for making clinical decisions.

## 1. Introduction

Obstructive sleep apnea (OSA) is a condition in which oxygen levels in the brain are reduced by the cessation of breathing during sleep, resulting in sleep disturbances [1]. OSA has been associated with the onset and worsening of lifestyle-related diseases such as hypertension, myocardial infarction, and diabetes. OSA can also lead to excessive daytime sleepiness, significantly increasing the risk of accidents [2]. Continuous positive airway pressure (CPAP) therapy remains the gold standard for the treatment of OSA in Europe and the United States. However, patients with mild-to-moderate OSA can also be treated using oral appliances (OA) [3].

In Japan, OA treatment is generally introduced in medical or dental clinics following polysomnography evaluation when CPAP is not available. The demand for OA has been increasing [4,5] because many patients prefer OA while traveling. In addition, some patients cannot tolerate CPAP.

OA can include mandibular advancement devices (MADs) and tongue stabilization devices [6]. In particular, monoblock MADs are commonly used to improve upper airway obstruction in Japan. Such devices function by moving the mandible forward, lifting the hyoid bone, and moving the soft palate forward via the palatoglossal arch, which connects the tongue and soft palate [7]. This mechanism promotes nasal breathing and creates an oral seal by preventing mouth opening and velopharynx occlusion [8]. However, monoblock MADs are not suitable for patients with OSA who exhibit nasal obstruction, since mouth breathing breaks the oral seal when nasal resistance is high [9]. In many cases, this represents a major obstacle that necessitates discontinuation of OA treatment [10]. Because previous reports have indicated that there are differences between subjective and objective measurements of nasal resistance, objective evaluations of nasal airflow are necessary [11]. Zeng et al. [12] demonstrated that high levels of nasal resistance measured using supine posterior rhinomanometry predicted poor treatment outcomes with mandibular advancement. Rhinomanometry is the standard technique for measuring nasal airway resistance through the measurement of nasal pressure and airflow. However, it is not readily available in dental sleep clinics. A recent study highlighted the efficacy of a small, lightweight device for easily assessing peak nasal inspiratory flow (PNIF) in patients with asthma and nasal obstruction [13]. Accordingly, we utilized this device to investigate awake nasal obstruction by recording PNIF in first-time visitors to our outpatient dental sleep clinic and examined the relationship between subjective and objective evaluations of nasal obstruction. The null hypotheses of the study state that (1) there is no difference in PNIF between OSA patients and the healthy participants, and (2) PNIF is not significantly correlated with AHI, SpO_2_min, Mallampati classification, NOSE or ESS scores.

## 2. Materials and Methods

### 2.1. Participants

Our study included 97 patients with OSA (65 men, mean age: 47.2 ± 15.2 years; 32 women, mean age: 51.8 ± 15.2 years) who had visited Nihon University School of Dentistry at Matsudo Hospital for OA from July 2019 to January 2020. The control group consisted of 105 adults (68 men, mean age: 34.2 ± 11.2 years; 37 women, mean age: 36.4 ± 11.0 years) with no nasal complaints as assessed by the Nasal Obstruction Symptom Evaluation (NOSE) scale and the PNIF (Table 1). Patients being treated currently for otorhinolaryngologic disorders, athletes such as sumo wrestlers and rugby players, patients with systemic diseases and restrictions to perform vigorous exercise, and patients who could not self-assess were excluded.

The study was approved by the Ethics Review Committee of Nihon University School of Dentistry at Matsudo (approval number EC-18-015: 27/09/2018). The study was performed according to the principles stated in the Declaration of Helsinki. All study participants provided written informed consent prior to inclusion in the study.

### 2.2. Sample Size Calculation

The estimated sample size was 91 based on the statistical power of 80% and a level of significance set at 5% to detect an effect size of 0.12 in the small range according to previously reported data [14]. The final estimated sample size was 96 when a dropout rate of 5% was added. The number of OSA patients seen in our outpatient dental sleep clinic over the 6-month period was 97. Accordingly, we recruited the same number of controls for the study.

### 2.3. Data Collection

The following variables were evaluated using medical records from the first visit in the OSA group: age, gender, body mass index (BMI), apnea-hypopnea index (AHI), minimum SpO_2_ (SpO_2_min), PNIF, NOSE scale scores, Mallampati classification, and Epworth Sleepiness Scale (ESS) scores. In the control group, the following variables were evaluated: PNIF, NOSE scores, and Mallampati classification.

### 2.4. PNIF Assessment

A portable PNIF meter (in-check DIAL, Clement Clarke International, Harlow, Essex, UK) was used to assess nasal function (peak inspiratory flow rate) in the present study. The PNIF assessment is an established, validated clinical tool for evaluating nasal obstruction [15]. Results obtained using this simple, yet reliable procedure correspond strongly with subjective assessment of nasal obstruction. The mean of three approved PNIF measurements for each patient was calculated at the initial visit, with the patient in a seated position with the head held parallel to the floor. When the measured value exceeded 1 SD, they were rested for 10 minutes and measured again.

### 2.5. NOSE Scale Scores

The NOSE scale was developed to assess the impact of nasal obstruction on the quality of life [16]. This scale contains five items (nasal congestion/stiffness, nasal blockage/obstruction, trouble breathing through nose, trouble sleeping, and inability to get enough air through the nose during exercise/exertion) assessed for the last one month and scored along a five-point scale (0 to 4), with 0 representing “not a problem” and 4 representing “a severe problem.” Total NOSE scores are calculated by multiplying the raw score by 5, with final scores ranging from 0 to 100 and classified as: 0, no obstruction; 5–25, mild obstruction; 30–50, moderate obstruction; 55–75, severe obstruction; and 80–100, extreme obstruction. The NOSE scale is a valid and reliable instrument [17,18].

### 2.6. Mallampati Classification

Mallampati scoring is a simple, noninvasive, inexpensive technique that involves visualization of the oropharynx. It is easy to learn and does not need any special equipment or setting. It has been used for more than two decades to assess the ease of intubation in anesthesiology [19]. The patients were asked to sit upright with the head positioned parallel to the floor, to open their mouth as widely as possible, and to protrude the tongue as much as possible. The observer sat opposite the patient at the level of the patient’s eye and inspected the pharyngeal structures of the patient with the help of a pen torch. The airway was then classified according to the structures visible, as follows: class I—soft palate, fauces, uvula, pillars; class II—soft palate, fauces, uvula; class III—soft palate, base of uvula; class IV—soft palate not visible at all. The Mallampati score has additional value in diagnosing OSA in adults according to The American Academy of Sleep Medicine [20].

### 2.7. Statistical Analysis

Independent *t*-tests were used to compare average PNIF measurements between the OSA and control groups. In addition, Levene’s test was used to examine the homoscedasticity of PNIF values. Pearson’s product–moment correlation coefficients were used to examine the relationship between basic clinical variables and nasal airflow assessments (PNIF and NOSE) in the OSA group. According to Cohen’s convention for the size of the effect for the Pearson correlation coefficient, an absolute value of *r* of 0.1 was classified as small, of 0.3 was classified as medium, and of 0.5 was classified as large [21]. *P*-values less than 0.05 were considered statistically significant. All statistical analyses were performed using SPSS for Windows version 20.0 software (SPSS, Chicago, IL, USA).

## 3. Results

### 3.1. Basic Clinical Data in the OSA Group

Table 1 shows the demographics, PNIF, NOSE scores, and Mallampati classification level from 105 healthy participants and 97 patients with OSA. In addition, data on AHI, SpO_2_min (%), and the ESS scores were collected for the OSA group (Table 1). The group had moderate sleep apnea with a mean AHI of 16.0 ± 9.2.

### 3.2. PNIF Results between OSA Group and Control Group

The mean PNIF values were significantly lower in the OSA group (101.3 ± 44.4 L/min) than in the control group (134.2 ± 31.5 L/min) (*p* < 0.001). The mean PNIF value remained significantly lower for both males and females in the OSA group than in the control group (*p* < 0.001) (Table 1).

Levene’s test for homoscedasticity yielded the following results for the patient and control groups: *F* = 17.64, *p* < 0.001. When the analyses were restricted to male or female participants, the *F* values were 11.73 (*p* < 0.001) and 15.62 (*p* < 0.001), respectively. The standard deviation was significantly larger in the OSA group than in the control group, regardless of gender.

### 3.3. NOSE and Mallampati Classification between the OSA and the Control Groups

The mean NOSE scores were significantly higher in the OSA group (29.1 ± 22.6 point) than in the control group (8.1 ± 5.5 point) (*p* < 0.001). Moreover, the mean Mallampati classification was significantly higher in the OSA group (3.7 ± 0.7) than in the control group (2.5 ± 0.6) (*p* < 0.001).

### 3.4. Correlations between Basic Clinical Variables and Nasal Obstruction Assessments in Patients with OSA

We did not observe a significant correlation between PNIF and AHI (*r* = −0.074, *p* = 0.468), PNIF and SpO_2_min (*r* = 0.030, *p* = 0.773), PNIF and ESS (*r* = 0.034, *p* = 0.741), PNIF and Mallampati Classification (*r* = -0.049, *p* = 0.631), PNIF and age (*r* = 0.034, *p* = 0.742), or PNIF and BMI (*r* = 0.081, *p* = 0.432). However, we observed a marginally significant negative correlation between PNIF and NOSE (*r* = −0.19, *p* = 0.061).

**Figure 1:**
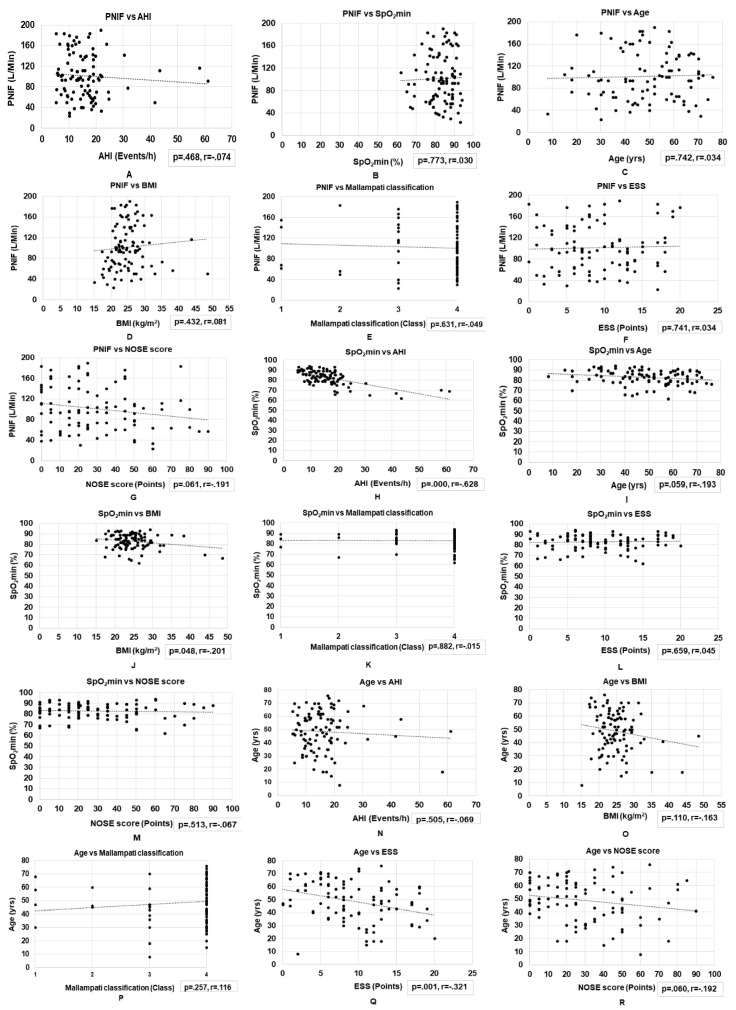

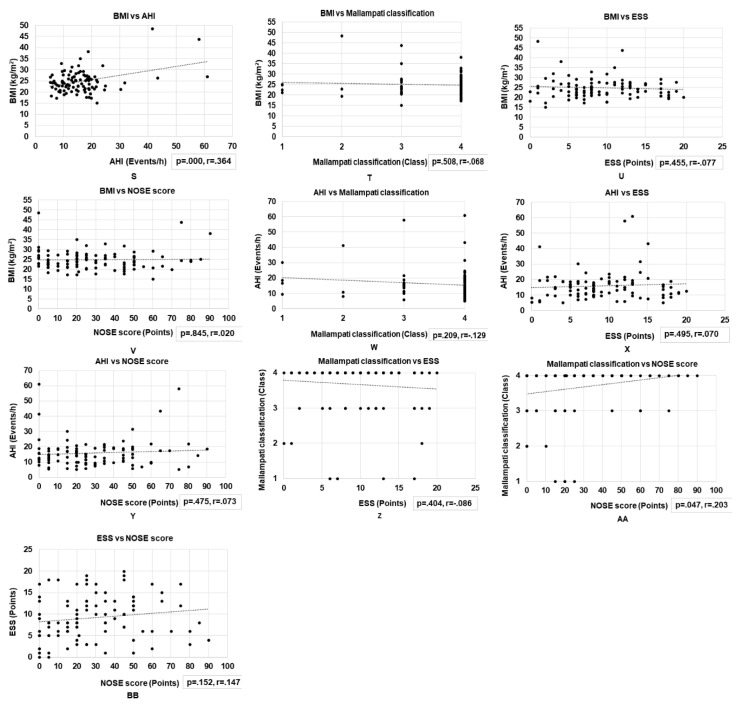
Correlations between basic clinical variables and nasal obstruction assessments in patients with obstructive sleep apnea. (**A**–**G**) Correlations between PNIF and AHI, SpO_2_min, age, BMI, Mallampati classification, ESS, and NOSE scores. (**H**–**M**) Correlations between SpO_2_min and AHI, age, BMI, Mallampati classification, ESS, and NOSE scores. (**N**–**R**) Correlations between age and AHI, BMI, Mallampati classification, ESS, and NOSE scores. (**S**–**V**) Correlations between BMI and AHI, Mallampati classification, ESS, and NOSE scores. (**W**–**Y**) Correlations between AHI and Mallampati classification, ESS, and NOSE scores. (**Z**–**AA**) Correlations between Mallampati classification and ESS and NOSE scores. (**BB**) The correlation between ESS vs. NOSE. AHI, apnea–hypopnea index; BMI, body mass index; ESS, Epworth Sleepiness Scale; NOSE, Nasal Obstruction Symptom Evaluation; PNIF, peak nasal inspiratory flow; SpO_2_min, minimum oxygen saturation.

We observed a weak yet significant positive correlation between NOSE and Mallampati Classification (*r* = 0.203, *p* = 0.047) and between AHI and BMI (*r* = 0.364, *p* < 0.01). In addition, we observed a significant negative correlation between AHI and SpO_2_min (*r* = −0.628, *p* < 0.01), between age and ESS (*r* = −0.321, *p* < 0.01), and between BMI and SpO_2_min (*r* = −0.20, *p* = 0.048). We observed a marginally significant negative correlation between age and SpO_2_min (*r* = −0.19, *p* = 0.059) and between age and NOSE (*r* = −0.19, *p* = 0.060) (Figure 1).

## 4. Discussion

To our knowledge, our study is among the first to objectively evaluate nasal airflow in a Japanese outpatient sleep dentistry clinic. Although OA and CPAP treatment can ease obstructive apneas, a nasal obstruction may preclude continuation with these treatments [10]. We, therefore, performed screening for nasal obstruction in OSA patients and compared that to a group of healthy control participants by means of objective PNIF assessments and the subjective NOSE scale. The patient group with OSA exhibited significantly lower PNIF values than healthy controls, suggestive of impaired nasal airflow. The subjective NOSE scores for the patient group bordered on moderate obstruction (mean 29.1 ± 22.6) and that for the healthy group on normal-mild obstruction (mean 8.1 ± 5.5).

In comparison to a previous multicenter, large-scale study of OA treatment in Japan [22], our study comprised of a large number of female patients, and our overall patient group tended to be younger. In addition, the mean AHI was lower among our outpatients than in previous large-scale studies, indicative of more patients with mild-to-moderate OSA in our study. These results are expected since our outpatient department specializes in sleep dentistry and patients with mild-to-moderate OSA are referred for OA treatment by nearby sleep clinics. Often, these patients are referred for OA therapy when they have discontinued CPAP treatment. The suburban location of our hospital may also explain the high percentage of female patients in our study.

Our findings for both healthy male and female participants are consistent with those of Ottaviano et al. [23] and Dor-Wojnarowska et al. [24] and confirm the higher PNIF values among men than women. The reason for the higher PNIF in males is the smaller nasal cavity found in females [25]. Differences in lung function may also explain these findings, as previous research has indicated that females have smaller lungs and narrower airways than males of the same age and BMI [26]. Thus, the differences in observed values between the sexes could depend on the difference in the size of the lungs and nasal cavity. Factors other than lung and nasal cavity size, such as an anatomical deviation in the nasal passage, should be considered.

In the OSA group, the PNIF findings obtained are consistent with those of Moxness et al. [14], who also reported lower values in the OSA compared with the control group. The Levene’s test further revealed that the standard deviation of PNIF values was greater in the OSA group than in the control group, suggestive of greater individual variation among patients. Moxness et al. [14] reported that the nasal cavity volume was significantly lower in the OSA group than in the healthy controls. In addition, a reduced response to treatment for congestion in the OSA group indicates a high bone-to-mucosa ratio in the inferior turbinate or an inflammatory cause of mucosal edema. Our outpatients may have these causes.

Although rhinomanometry is the gold standard test for assessing nasal airflow [27] and is highly accurate, it is not available in sleep dental clinics, and patients must visit an otolaryngologist for the assessment, making it impractical with regard to time and cost. On the other hand, PNIF measurement is a simple, objective assessment of inspiratory nasal airflow. The ease of obtaining objective data related to nasal airflow in sleep dentistry clinics may provide a means of assessing the degree of nasal obstruction prior to OA/CPAP treatment and thus may help improve adherence to these treatments. However, the daytime PNIF measure does not correlate with any of the sleep apnea variables; it bears no relationship with the AHI or ESS scores, suggesting that PNIF cannot be used to assess treatment outcomes with OA. This measurement was taken with the subjects in an upright posture. A supine PNIF measurement might be strongly associated with AHI, as it is expected to be higher given the reduced pharyngeal diameter while lying down [28]. Thus, supine PNIF would have been a more appropriate measurement for this study.

In the patient group, as expected, the sleep apnea variables were highly correlated, with a high AHI and high BMI predicting a low SpO_2_ and increasing age predicting lower sleepiness, SpO_2,_ and lower subjective nasal obstruction. These findings are in line with previous findings of a positive correlation between AHI and BMI [29] and a negative correlation between BMI and the lowest SpO_2_ [30], between AHI and SpO_2_min [31], and between age and ESS scores [32].

The NOSE score and Mallampati classification were significantly higher in patients with OSA than in the healthy controls and lower in females than males. Kale et al. [33] had similarly reported a significantly higher Mallampati classification in the OSA group than in the healthy control group. These subjective evaluations confirm that OSA patients are conscious of their nasal obstruction.

The NOSE scores correlated positively with the Mallampati classification. The NOSE scale, developed and validated by Stewart et al. [15], as a measure of nasal obstruction, is also useful in patients with OSA [34]. Previous studies have reported that high Mallampati grades in patients with nasal obstruction may worsen OSA severity [35], making assessments of Mallampati grade valuable. In addition, Yagi et al. [36] reported that the Mallampati grade predicted severity of OSA linked to anatomical/morphological characteristics in Japanese patients. This may explain the correlation between the NOSE scale scores and Mallampati classification in our patients with mild-to-moderate symptoms. The fact that there was a significant, albeit marginal, negative correlation between PNIF and NOSE (*p* = 0.061, *r* = −0.191) suggests that the perception of nasal obstruction was accurate. The marginal significance may be explained by the small sample size, and we would like to increase the number of participants in future studies.

The present study has some limitations. First, because of the large number of male OSA patients, we saw more male patients than female patients during the 6-month period. In the future, we would like to extend the survey period and derive new data when the numbers of men and women are equal. Second, there was a larger proportion of patients with mild-to-moderate OSA. Third, we measured PNIF in the upright rather than the supine position. Future studies should examine PNIF in the supine position with or without OA for the same patient and systematically evaluate if objective assessments of awake PNIF can help in evaluating the effectiveness of OA/CPAP treatment.

## 5. Conclusions

Lower PNIF values and higher NOSE scores suggested impaired nasal airflow in the OSA group. However, daytime PNIF measurement bears no relationship to AHI and the severity index of sleep apnea and cannot be used alone as an objective screening tool for predicting the suitability of OA treatment in sleep dentistry outpatients. It can, however, be used as an adjunct for making clinical decisions.

## Figures and Tables

**Table 1:** The characteristics of OSA patients and controls.

	Control	OSA	*p* Value
N	105 (100)	97 (100)	
Males, n (%)	68 (64.8)	65 (67.0)	
Females, n (%)	37 (35.2)	32 (33.0)	
Age (years)	35.0 ± 11.1	48.7 ± 15.3	
BMI (kg/m^2^)	22.6 ± 2.0	24.8 ± 5.1	
AHI (/h)	N/A	16.0 ± 9.2	
Mild, n (%)	N/A	49 (50.5)	
Moderate, n (%)	N/A	42 (43.3)	
Severe, n (%)	N/A	6 (6.2)	
SpO_2_min (%)	N/A	82.8 ± 7.1	
ESS (point)	N/A	9.2 ± 5.0	
PNIF (L/min)	134.2 ± 31.5	101.3 ± 44.4	<0.001
Male (L/Min)	143.4 ± 33.6	118.0 ±45.5	<0.001
Female (L/Min)	117.4 ±17.7	81.1 ±34.3	<0.001
NOSE questionnaire(point×5)	8.1 ± 5.5	29.1 ± 22.6	<0.001
Mallampati (Class)	2.5 ± 0.6	3.7 ± 0.7	<0.001

N: number; BMI: body mass index; AHI: apnea–hypopnea Index; SpO_2_min: minimum SpO_2_; ESS: Epworth Sleepiness Scale; PNIF: peak nasal inspiratory flow; NOSE: Nasal Obstruction Symptom Evaluation. Sleep apnea severity was classified as mild: 5≤AHI<15, moderate: 15≤AHI<30, and severe AHI≥30.
